# Complex Gastrointestinal and Endocrine Sources of Inflammation in Schizophrenia

**DOI:** 10.3389/fpsyt.2020.00549

**Published:** 2020-06-16

**Authors:** Emily G. Severance, Faith Dickerson, Robert H. Yolken

**Affiliations:** ^1^Stanley Division of Developmental Neurovirology, Department of Pediatrics, Johns Hopkins University School of Medicine, Baltimore, MD, United States; ^2^Sheppard Pratt Health System, Towson, MD, United States

**Keywords:** gut–brain axis, immune system, microbiome, bacterial translocation, metabolic syndrome

## Abstract

A low level, inflammatory phenotype is prevalent in individuals with schizophrenia, but the source of this inflammation is not known. Studies of the gut–brain axis indicate that this inflammation may be related to the translocation of intestinal microbes across a permeabilized gut–vasculature barrier. In addition, studies of the endocrine system support that this inflammation may derive from effects of stress hormones and metabolic imbalances. Gastrointestinal (GI) and endocrine conditions are not mutually exclusive, but rather may have additive effects to produce this inflammatory phenotype in schizophrenia. Here, we examined a series of plasma biomarkers used to measure general inflammation and presumably microbial, gut-derived inflammation in 409 individuals with schizophrenia: c-reactive protein (CRP), lipopolysaccharide-binding protein (LBP), soluble CD14 (sCD14), and IgG antibodies to *S. cerevisiae*, bovine milk casein, and wheat gluten. Individuals were stratified according to whether or not they had a comorbid GI or endocrine condition, both, or neither. In multivariate regression models, the presence of GI and endocrine conditions was additive for the GI-based marker, LBP, with significant associations only when both conditions were present compared to when both conditions were absent (OR = 2.32, 95^th^% CI 1.05–5.13, p < 0.03). In contrast, the marker of general inflammation, CRP, was strongly associated with primarily endocrine conditions (OR = 3.64, 95th% CI 1.35–9.84, p < 0.05). Overall associations were largely driven by the GI condition, gastroesophageal reflux disease (GERD), and by the endocrine condition, obesity. In univariate comparisons, *S. cerevisiae* IgG levels were significantly elevated only in persons with GI conditions (p < 0.02), whereas antibodies to the food antigens were elevated in the presence of either or both conditions (p < 0.005–0.04). More severe psychiatric symptoms were associated only with GI conditions (p < 0.01–0.04). In conclusion, both GI and endocrine abnormalities may contribute to inflammation in schizophrenia, sometimes independently and sometimes as part of interactions which may represent complex integrated pathways. The accumulating evidence for multisystem inflammation in schizophrenia may lead to the development of new strategies to prevent and treat this devastating disorder.

## Introduction

Schizophrenia is a serious psychiatric disorder of unknown etiology. The disorder is thought to be the product of genetic and environmental interactions consistent with involvement of the immune system. Genome-wide-association-studies point to schizophrenia risk loci in the major-histocompatibility-complex region of chromosome 6 where many immune-related genes are located (1). A susceptibility locus for schizophrenia, complement C4, is of interest since it is a component of the immune system and is also involved in synaptic pruning in the brain (2, 3). Environmental and epidemiological studies indicate that people with schizophrenia have increased rates of exposure to pathogens and other antigens (4, 5). A reported phenotype in these individuals is a pervasive low level inflammation of unknown origin (6, 7).

Immune system homeostasis is principally established and regulated by the GI mucosa and its community of resident microbiota (8, 9). When this balance is upset, for example by stress, toxins, infections, antibiotics or genetic susceptibility, a toxic cycle of inflammation, microbial translocation and dysbioses ensue. In the literature of schizophrenia, there is a long history that GI disturbances are part of the pathophysiology of this disorder, with many disturbances inflammatory in nature. Related reports predate the advent of modern antipsychotics, suggesting that this GI inflammation is not simply the result of cholinergic effects from agents such as clozapine (10).

The endocrine system is another postulated source for the chronic inflammation associated with schizophrenia. Especially implicated are certain conditions including metabolic syndrome, obesity, and diabetes as well as basic differences in disease pathophysiology between males and females (11–13). These conditions are often the result of antipsychotics that slow metabolism and contribute to weight gain and a disrupted metabolic state. Metabolic syndrome may occur in up to 50% of people with schizophrenia either through a direct modulation of insulin-associated tissue, glucose metabolism or increased production of adipose tissue (14). Another endocrine-based candidate is the end product of the hypothalamus–pituitary-axis (HPA), the stress hormone, cortisol. Alterations of the cortisol awakening response is a proposed risk factor for the development of schizophrenia (15).

Research on schizophrenia and other psychiatric disorders has begun to focus on organ systems outside of the brain to determine if mechanisms involved in the periphery and as part of a whole-body approach might offer fresh perspectives and insight regarding disease etiology and treatment. Given the high prevalence of these comorbid conditions and the pervasive inflammatory phenotype in schizophrenia, we undertook to ascertain the relative contributions of GI and endocrine disturbances to the inflammatory pathophysiology of schizophrenia. There is currently uncertainty regarding how these conditions are related to each other and to what degree each may be the source of inflammation in schizophrenia. Thus, further complicating this issue is the uncertainty regarding the specificity of serum or plasma biomarkers as proxies for GI and endocrine levels of inflammation. Blood biomarkers typically used to measure gut-derived microbial translocation include LBP and sCD14, but the specificity of these markers for GI processes have been questioned (16, 17). Another biomarker of microbial translocation, antibodies directed against *S. cerevisiae*, is one component of a serological panel used clinically to diagnose the inflammatory bowel disorder, Crohn’s disease (18–20). Similarly, antibodies against food-derived proteins such as wheat gluten and milk casein are used to diagnose food antigen sensitivities and for wheat gluten, a clinical diagnosis of Celiac disease (21, 22). In this study, we review data from individuals with schizophrenia who had reported or had in their medical record information about the presence of GI or endocrine conditions comorbid with their psychiatric disorder. We measured plasma levels of systemic inflammation using CRP and compared these to measures of presumably GI-related biomarkers in individuals with schizophrenia with and without GI or endocrine disorders.

## Materials and Methods

### Study Population

Study participants were recruited at Sheppard Pratt Health System located in Baltimore, MD, U.S.A. as part of an ongoing schizophrenia cohort. We reviewed the study database to include individuals with schizophrenia for whom we had information regarding the presence or absence of GI and endocrine conditions. The original collection of this information was carried out as a standard assessment in our studies. A total of 409 individuals with schizophrenia were identified. These individuals were stratified into four groups: (1) GI negative and endocrine negative (GI−/endocrine−); (2) GI positive and endocrine negative (GI+/endocrine−); (3) GI negative and endocrine positive (GI−/endocrine+); (4) GI positive and endocrine positive (GI+/endocrine+). The classification of conditions was done systematically by review of medical records and patient interviews performed by the research nurse. Conditions queried were based on past studies indicating their presence in the population of individuals which compose the study groups. As part of the research assessment, participants were queried about the presence or absence of specific disorders which were organized by body system, as described previously (23). GI conditions included constipation, Crohn’s disease, diarrhea, diverticulitis, GI bleeding, gastritis, GERD, irritable bowel syndrome, lactose intolerance, stomach cancer, surgical GI procedures, and ulcers. Endocrine conditions included Diabetes Mellitus (Type I and Type II), glucose-6-phosphate dehydrogenase (G6PD) enzyme deficiency, gynecomastia, hyperglycemia, hypernatremia, obesity, premenstrual dysphoric disorder, rhabdomyolysis, and thyroid problems. The prevalence of GI and endocrine conditions is listed in **Table 1**. For statistical comparisons described below, the comparison control group includes those individuals with schizophrenia who were GI−/endocrine−.

**Table 1:** Prevalence of comorbid GI and endocrine conditions.

	n	%
**Gastrointestinal**		
Constipation	86	29.55%
Crohn’s Disease	1	0.34%
Diarrhea	26	8.93%
Diverticulitis	3	1.03%
GI bleeding	1	0.34%
Gastritis	6	2.06%
GERD/acid reflux	120	41.24%
Irritable bowel syndrome	7	2.41%
Lactose intolerance	3	1.03%
Stomach cancer	2	0.69%
Surgical GI procedures	22	7.56%
Ulcers	14	4.81%
		
**Endocrine**		
Diabetes	63	23.60%
Glucose-6-Phosphate Dehydrogenase (G6PD) Enzyme Deficiency	7	2.62%
Gynecomastia	2	0.75%
Hyperglycemia	2	0.75%
Hypernatremia	1	0.37%
Obesity	155	58.05%
Premenstrual Dysphoric Disorder	3	1.12%
Rhabdomyolysis	1	0.37%
Thyroid problems	33	12.36%

Diagnostic methods were described previously (24, 25). Individuals received DSM-IV-TR diagnoses of schizophrenia, schizophreniform disorder, or schizoaffective disorder (26). Individuals were between the ages of 18 and 65. Cognitive functioning was evaluated with the Repeatable Battery for the Assessment of Neuropsychological Status (RBANS) Form A (27) and psychiatric symptoms rated with the Positive and Negative Syndrome Scale (PANSS) (28).

These studies were approved by the Institutional Review Boards (IRB) of the Sheppard Pratt Health System and the Johns Hopkins Medical Institution following established guidelines. All participants provided written informed consent after study procedures were explained. This research was performed in accordance with The Code of Ethics of the World Medical Association (Declaration of Helsinki) for experiments involving humans.

### Biomarker Data

For all participants, a blood sample was drawn from which were measured CRP, LBP, sCD14, *Saccharomyces cerevisiae* IgG, bovine milk casein IgG, and wheat gluten IgG. Biomarker positivity was defined based on quantitative levels of these markers in healthy controls. Biomarker values in the schizophrenia population which exceeded the 90th percentile of healthy control values were considered seropositive. Individuals who were considered healthy controls were those without a history of psychiatric disorder based on interviews carried out with the Structured Clinical Interview for DSM-IV Axis I Disorders Non-Patient Edition (29). Methods and analyses reporting psychiatric case and control levels and cut-off seropositivity values of these biomarkers were previously described (25, 30–34). In brief, exclusion criteria for both cases and controls included: mental retardation; clinically significant medical disorder that would affect cognitive performance; any history of intravenous substance abuse or a primary diagnosis of substance abuse or substance dependence. Active substance misuse was considered an additional exclusion criterion for controls. Comorbid GI and endocrine conditions were not exclusion criteria for cases or controls. The healthy control group was composed of 311 individuals of mean age 32+/−0.63 years, 61.09% female and were 64.31% Caucasian.

### Data Analyses

Chi-square analyses were used to detect significant differences in categorical variables among comorbidity groups. ANOVAs with *post-hoc* Sidak analyses and t-tests were used to identify mean differences between groups for continuous variables. Multivariate logistic regression models were used to assign odds ratios for biomarker positivity associations with GI/endocrine groups and to evaluate the interaction between GI and endocrine variables. All multivariate analyses included the covariates of age, sex, race, cigarette smoking, and maternal education as a proxy for socioeconomic status. P-values of <0.05 are listed; however, Bonferroni correction of multiple comparisons would designate more robust associations for those p-values that are <0.008.

## Results

### Characteristics of the Study Population

As described in the *Methods*, participants in this study were divided into four groups according to whether or not a comorbid GI or endocrine condition was present. The characteristics of these groups are shown in **Table 2**. GI conditions were present in 58.1% females and 48.7% males, and endocrine conditions were present in 64.2% females and 50.6% males. Females were more likely to have both GI and endocrine disturbances, whereas males were more likely to have neither (chi-square = 9.19, p < 0.03). Participants with both GI and endocrine disturbances also were older than those who had neither type of condition (ANOVA, F = 5.00, p < 0.001). There were no significant differences between the four GI and endocrine groups in terms of race, maternal education, cigarette smoking or current antipsychotic, anticholinergic or antibiotic medications (**Table 2**).

**Table 2:** Characteristics of the sample by GI and endocrine comorbidity status.

	GI−/Endoc−^1^	GI+/Endoc−	GI−/Endoc+	GI+/Endoc+	Total
	n (%)	n (%)	n (%)	n (%)	n
All	107 (26.16)	75 (18.34)	89 (21.76)	138 (33.74)	409
Female	27 (18.24)	26 (17.57)	35 (23.65)	60 (40.54)^2^	148
Male	80 (30.65)	49 (18.77)	54 (20.69)	78 (29.89)	261
Age (mean years + SE)	34.92 + 1.22	37.81 + 1.52	37.50 + 1.46	41.20 + 1.02^3^	409
Race					
Caucasian	45 (22.96)	39 (19.90)	37 (18.88)	75 (38.27)	196
Non-Caucasian	62 (29.11)	36 (16.90)	52 (24.41)	63 (29.58)	213
Maternal education (mean years + SE)	2.39 + 0.06	2.39 + 0.09	2.25 + 0.08	2.44 + 0.07	409
Cigarette smoker	64 (26.02)	49 (19.92)	56 (22.76)	77 (31.30)	246
Medications					
Aripiprazole	9 (19.57)	5 (10.87)	12 (26.09)	20 (43.48)	46
Clozapine	12 (19.05)	12 (19.05)	12 (19.05)	27 (42.86)	63
Olanzapine	24 (33.80)	16 (22.54)	13 (18.31)	18 (23.35)	71
Quetiapine	10 (20.83)	10 (20.83)	7 (14.58)	21 (43.75)	48
Risperidone	31 (28.44)	23 (21.10)	24 (22.02)	31 (28.44)	109
Ziprasidone	5 (22.73)	4 (18.18)	4 (18.18)	9 (40.91)	22
Anticholinergics	33 (21.85)	32 (21.19)	36 (23.84)	50 (33.11)	151
Antibiotics	2 (22.22)	5 (55.56)	0 (0.00)	2 (22.22)	9
Comorbid GI condition^4^					
GERD	0 (0.00)	34 (28.33)	0 (0.00)	86 (71.67)	120
Constipation	0 (0.00)	26 (30.23)	0 (0.00)	60 (69.77)	86
Diarrhea	0 (0.00)	4 (15.38)	0 (0.00)	22 (84.62)	26
Comorbid endocrine condition					
Obesity	0 (0.00)	0 (0.00)	62 (40.00)	93 (60.00)	155
Diabetes	0 (0.00)	0 (0.00)	21 (33.33)	42 (66.67)	63
Thyroid	0 (0.00)	0 (0.00)	3 (9.09)	30 (90.91)	33
Body mass index (mean score + SE)	25.2 + 0.39	25.75 + 0.48	32.79 + 0.69	34.69 + 0.68	386^5^

^1^Gastrointestinal (GI), Endocrine (Endoc), Negative (−), Positive (+).

^2^Female vs male differences among groups. Refer to text for details. Females were more likely to have both conditions and males neither: chi-square = 9.19, p < 0.03.

^3^Participants with both conditions were older than those who had neither: ANOVA, F = 5.00, p < 0.001.

^4^Comorbid conditions are listed for descriptive purposes only; prevalence is expected to vary between groups.

^5^Data not available for complete sample of 409.

### Biomarker Levels as Continuous Variables

Plasma levels of six biomarkers typically used as measures of GI and systemic inflammation were quantified, and differences relative to the non-GI/non-endocrine group are depicted in **Table 3**. Plasma levels of the general biomarker of inflammation, CRP, were significantly elevated in the GI−/endocrine+ and GI+/endocrine+ groups compared to the group without these conditions (ANOVA, F = 8.99, p < 0.0001). Plasma levels of LBP were significantly elevated in the GI−/endocrine+ and GI+/endocrine+ groups compared to the GI−/endocrine-free (ANOVA, F = 7.74, p < 0.0001). IgG antibody levels directed against the yeast *S. cerevisiae* and against the food antigens, bovine milk casein and wheat gluten, were elevated in the GI+/endocrine− groups with some variation according to sex as shown in **Table 3**. Antibody levels directed against the food antigens were also elevated in the GI−/endocrine+ and GI+/endocrine+ groups, again with some variation as shown in **Table 3**. Plasma levels of sCD14 were not significantly different between groups.

**Table 3:** Comparison of biomarker levels by sex and by GI and endocrine comorbidity status.

Biomarker		GI+/Endoc−	GI−/Endoc+	GI+/Endoc+
C-Reactive Protein	All	NS	p < 0.0002	p < 0.0001
	Female	NS	p < 0.03	p < 0.005
	Male	p < 0.03	p < 0.002	p < 0.0003
LPS-Binding Protein	All	NS	p < 0.001	p < 0.0001
	Female	NS	p < 0.007	p < 0.005
	Male	NS	p < 0.03	p < 0.001
Soluble CD14	All	NS	NS	NS
	Female	NS	NS	NS
	Male	NS	NS	NS
*Saccharomyces cerevisiae* IgG	All	p < 0.02	NS	NS
	Female	NS	NS	NS
	Male	p < 0.02	NS	NS
Bovine Casein IgG	All	NS	NS	NS
	Female	NS	NS	NS
	Male	p < 0.04	p < 0.03	ns
Wheat Gluten IgG	All	p < 0.03	p < 0.02	p < 0.03
	Female	NS	NS	NS
	Male	NS	p < 0.005	p < 0.01

**Figure 1:**
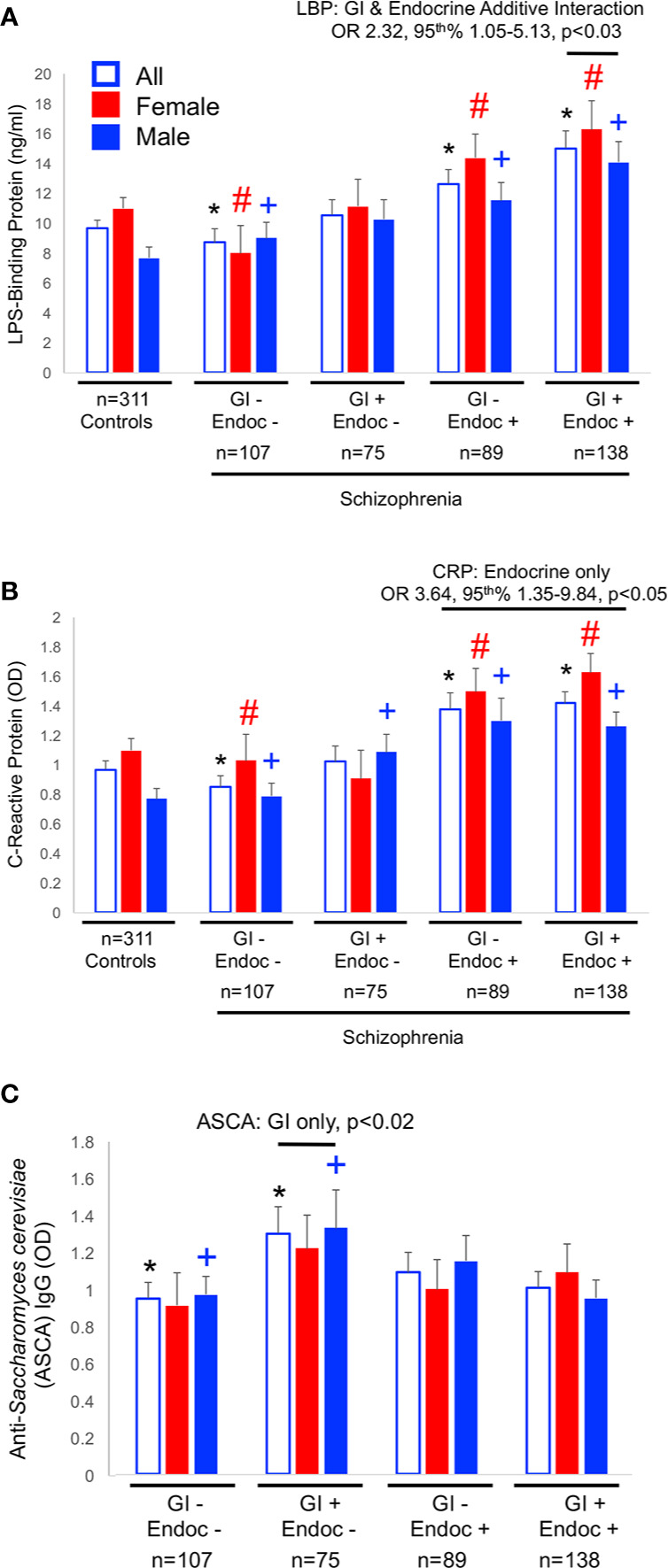
Biomarker levels for LBP **(A)**, CRP **(B)** and ASCA **(C)** are shown. Significant differences in levels from univariate comparisons are marked with an asterisk (*) for all members, with a pound sign (#) for females and a plus (+) for males. Data for multivariate comparisons were significant for LBP and CRP. Significant odds ratios (OR) and 95^th^% confidence interval (CI) are shown for LBP and CRP. LBP associations were a function of both GI and endocrine disturbances, CRP of endocrine only, and ASCA of only GI disturbances.

GI−/Endoc− is the comparison group. P-values were generated from T-tests so that results are comparable across groups and biomarkers. CRP and LBP were additionally significant following ANOVAs and multivariate comparisons and are reported in the main text and **Figure 1**.

### Biomarker Positivity as Categorical Variables in Multivariate Models

As described above, the strongest associations of biomarker levels with the GI/endocrine groups were found for CRP and LBP. Thus, we tested whether or not the GI and endocrine conditions were interactive in multivariate logistic regression models that included age, sex, race, cigarette smoking, maternal education, and the interactive GI/endocrine variable. Relative levels of these biomarkers among GI/endocrine groups are depicted in **Figure 1**. While neither GI nor endocrine disturbances were individually associated with LBP in multivariate models, GI and endocrine conditions were additive for associations with LBP positivity (**Figure 1A**: GI+/endocrine+, OR = 2.32, 95^th^% CI 1.05–5.13, p < 0.03). In contrast, GI and endocrine disturbances were not additive for CRP, but significant associations with CRP positivity were driven by endocrine disturbances (**Figure 1B**: endocrine only, OR = 3.64, 95th% CI 1.35–9.84, p < 0.05). For comparison, IgG antibodies to *S. cerevisiae* are included in **Figure 1** to illustrate the patterns of a biomarker that putatively reflect only GI-based disturbances (**Figure 1C**). This *S. cerevisiae* IgG association was not statistically significant in the multivariate models.

#### Contribution of Specific GI and Endocrine Conditions to Biomarker Associations

As shown in **Table 1**, the three most prevalent GI conditions in this schizophrenia population were GERD (41.24%), constipation (29.55%), and diarrhea (8.93%). The three most prevalent endocrine conditions were obesity (58.05%), diabetes (23.60%), and thyroid problems (12.36%). The prevalence of these conditions within the broader GI and endocrine groups is listed in **Table 2**. Body mass index was elevated in the endocrine positive groups compared to those who had neither GI nor endocrine conditions (ANOVA, F = 62.06, p < 0.0001). Associations of biomarker levels as continuous variables with specific GI and endocrine conditions are depicted in **Table 4**. For GI variables, individuals with GERD had significantly elevated levels of CRP (p < 0.0003), LBP (p < 0.0002) and sCD14 (p < 0.004) compared to those without GERD. For people with constipation, only wheat gluten IgG levels were significantly elevated compared to those without constipation (p < 0.03). Levels of LBP (p < 0.02), sCD14 (p < 0.02) and *S. cerevisiae* IgG levels (p < 0.04) were all elevated in individuals with diarrhea compared to those without. For the endocrine variables, LBP was elevated in individuals who were obese (p < 0.0001), had diabetes (p < 0.01) or thyroid problems (p < 0.04) compared to those who did not have these conditions. CRP levels were elevated in those who were obese (p < 0.0001) or who had diabetes (p < 0.03). In individuals who had thyroid problems, wheat gluten IgG levels were elevated (p < 0.03).

**Table 4:** Associations of biomarkers with specific GI and endocrine conditions.

GI conditions		GERD-	GERD+	p-value	Constipation-	Constipation+	p-value	Diarrhea-	Diarrhea+	p-value
	n	289	120		323	86		383	26	
C-Reactive Protein	Mean + SE	1.09 + 0.06	1.45 + 0.08	p < 0.0003	1.16 + 0.06	1.33 + 0.10	NS	1.19 + 0.05	1.33 + 0.14	NS
LPS-Binding Protein	Mean + SE	10.64 + 0.56	15.00 + 1.27	p < 0.0002	11.57 + 0.59	13.2 + 1.42	NS	11.63 + 0.55	16.14 + 2.95	p < 0.02
Soluble CD14	Mean + SE	1.29 + 0.11	1.85 + 0.20	p < 0.004	1.42 + 0.11	1.56 + 0.23	NS	1.40 + 0.10	2.26 + 0.42	p < 0.02
*Saccharomyces cerevisiae* IgG	Mean + SE	1.04 + 0.06	1.19 + 0.11	NS	1.05 + 0.06	1.22 + 0.13	NS	1.06 + 0.05	1.43 + 0.27	p < 0.04
Bovine Casein IgG	Mean + SE	1.01 + 0.06	0.96 + 0.10	NS	1.02 + 0.06	0.90 + 0.10	NS	0.99 + 0.05	1.15 + 0.25	NS
Wheat Gluten IgG	Mean + SE	1.00 + 0.07	0.97 + 0.10	NS	0.94 + 0.06	1.19 + 0.13	p < 0.03	1.00 + 0.06	0.89 + 0.09	NS
										
**Endocrine conditions**		**Obesity−**	**Obesity+**	**p-value**	**Diabetes−**	**Diabetes+**	**p-value**	**Thyroid−**	**Thyroid+**	**p-value**
	n	254	155		346	63		376	33	
C-Reactive Protein	Mean + SE	0.96 + 0.05	1.58 + 0.09	p < 0.0001	1.16 + 0.05	1.41 + 0.12	p < 0.03	1.19 + 0.05	1.30 + 0.13	NS
LPS-Binding Protein	Mean + SE	9.95 + 0.60	15.14 + 1.04	p < 0.0001	11.40 + 0.59	14.75 + 1.50	p < 0.01	11.63 + 0.56	15.21 + 2.56	p < 0.04
Soluble CD14	Mean + SE	1.28 + 0.11	1.74 + 0.18	p < 0.01	1.47 + 0.11	1.38 + 0.21	NS	1.43 + 0.10	1.75 + 0.44	NS
*Saccharomyces cerevisiae* IgG	Mean + SE	1.13 + 0.07	1.01 + 0.08	NS	1.10 + 0.06	0.98 + 0.12	NS	1.08 + 0.05	1.13 + 0.22	NS
Bovine Casein IgG	Mean + SE	0.98 + 0.06	1.03 + 0.09	NS	1.00 + 0.06	0.96 + 0.133	NS	0.99 + 0.05	1.04 + 0.18	NS
Wheat Gluten IgG	Mean + SE	0.95 + 0.06	1.05 + 0.11	NS	1.01 + 0.06	0.89 + 0.08	NS	0.96 + 0.05	1.35 + 0.36	p < 0.03

Negative (−), Positive (+).

P-values were generated from T-tests.

We then used multivariate models to detect interactions of specific GI and endocrine conditions with the biomarkers. We found that the earlier interactive associations of the broadly grouped conditions were largely driven by the GI variable, GERD, and the endocrine variable, obesity. For example, LBP was independently associated with both GERD and obesity (GERD: OR = 2.42, 95th% CI 0.99–5.95, p < 0.05; Obesity: OR = 3.40, 95th% CI 1.58–7.31–31.71, p < 0.002), as well as to the additive interaction of these terms (OR = 4.64, 95th% CI 2.03–10.65, p < 0.001). Further, LBP was independently associated with diarrhea (OR = 7.99, 95th% CI 2.01–31.71, p < 0.003), but this term did not interact with obesity or other endocrine variables. CRP was strongly associated with obesity (OR = 5.36, 95th% CI 2.45–11.73, p < 0.002) but not to an interaction of obesity with GERD. We also found that the interaction of the GI variable, constipation, with the endocrine variable, thyroid problems, was significantly associated with gluten antibody positivity (OR = 167.34, 95^th^% CI 2.58–10,836.33, p < 0.016). Neither variable alone was associated with these gluten antibodies.

### Cognitive Functioning and Psychiatric Symptoms

Finally, we evaluated the association between the GI and endocrine groups and the level of cognitive functioning and psychiatric symptom severity based on scores from RBANS and PANSS modules. Higher scores on the PANSS total symptom score and the PANSS positive symptom score were found for the GI+/endocrine− group compared to the GI−/endocrine− group (T-test t range = −2.30 to −1.81, p-value range <0.01–0.04; **Table 5**). Specific GI or endocrine conditions were not significantly associated with PANSS scores. There were no significant differences in RBANS scores among the broader GI and endocrine groups; however, lower scores were observed for those who were GERD positive compared to those who were GERD negative (T-test t = 1.81, p < 0.04).

**Table 5:** Cognitive and psychiatric symptom scores according to gut and endocrine conditions.

	GI−/Endoc− Score + SE	GI+/Endoc− Score + SE	p-value	GI−/Endoc+ Score + SE	p-value	GI+/Endoc+ Score + SE	p-value
PANSS^1^ Total	76.57 + 1.33	80.31 + 1.57^3^	p < 0.04	77.47 + 1.47	NS	77.26 + 1.16	NS
Positive	18.99 + 0.48	20.73 + 0.60^4^	p < 0.01	19.63 + 0.56	NS	19.95 + 0.46	NS
Negative	21.48 + 0.51	21.17 + 0.38	NS	21.28 + 0.48	NS	20.38 + 0.37	NS
RBANS^2^	64.54 + 1.20	65.07 + 1.38	NS	64.47 + 1.26	NS	64.82 + 0.98	NS

^1^PANSS refers to the Positive and Negative Syndrome Scale.

^2^RBANS refers to the Repeatable Battery for the Assessment of Neuropsychological Status.

^3^GI−/Endoc− is the comparison group; T-test t value = −1.81, p < 0.04.

^4^T-test t value = −2.30, p < 0.01.

## Discussion

In this clinical study of inflammatory biomarkers, we found significant interplay between GI and endocrine systems in schizophrenia and no single biomarker reflected a sole GI or endocrine affinity, with the possible exception of *S. cerevisiae* antibodies with GI conditions. The most robust associations were found for LBP and CRP. We found that the presence of both GI and endocrine conditions was additive for the marker, LBP, and these associations were particularly evident when the broader categories of GI and endocrine disorders were broken down into specific conditions such as GERD and obesity. Plasma LBP has been traditionally considered as a marker of bacterial translocation which specifically detects and binds any circulating bacterial-derived LPS endotoxin. LBP associations with endocrine conditions suggest its modulation by hormonal and metabolic factors such as obesity, diabetes and thyroid dysfunction. Indeed, the gut microbiome and its metabolic products, some of which are hormonal, are thought to mediate behavioral responses in part through interactions with neuroendocrine pathways that link the gut and central nervous system (35, 36).

We found a significant association between CRP, a general marker for inflammation, primarily with endocrine conditions and to a lesser extent in univariate models with GI conditions. Antibodies directed against the dietary yeast, *S. cerevisiae*, represented the only biomarker that was associated exclusively with a GI condition. Thus, results from this study support that antibodies to *S. cerevisiae*, a marker elevated in inflammatory bowel diseases (18), may be in fact a specific measure of perturbed GI conditions. Even antibodies directed against the food antigens were associated with both GI and endocrine conditions with some variations according to sex. Interestingly, the interaction of constipation and thyroid problems was significantly associated with antibodies to wheat gluten. The other marker of bacterial translocation, sCD14, showed few significant differences in levels among GI/endocrine groups suggesting an alternative mechanism to explain its associations with schizophrenia, perhaps related to monocyte activation (17). Although sCD14 and LBP markers showed a low level of correlation in our study (data not shown), the lack of corresponding GI and endocrine group differences for sCD14 further supports its role in inflammatory pathways that do not necessarily impact LBP.

More severe psychiatric symptoms, and particularly positive symptoms, were significantly associated with GI and not endocrine conditions. Studies of the gut–brain axis increasingly demonstrate a role for the microbiome and downstream pathological effects related to inflammation when this microbial community is in dysbiosis. In schizophrenia, case–control differences in the blood biomarkers examined here suggest that a low-grade peripheral inflammation is related to the translocation of gut-based microbes and metabolic products across compromised gut-associated vascular barriers into systemic circulation. This low-grade systemic inflammation, in turn, is thought to produce similar permeability at the blood-brain barrier, thus providing a mechanism by which gut-based products might enter the central nervous system and alter brain functioning (4, 37, 38).

The extent to which our results can be extrapolated is limited by several factors. This study is cross-sectional which prevents us from knowing if levels of these markers fluctuate over time or from making cause and effect interpretations. Furthermore, our control group comparison upon which seropositivities were designated may have included individuals with GI or endocrine conditions; thus, seropositivity cut-offs may be over-estimated and associated with a possible increase in false-negatives in the case group. Our subgroup stratification reduces the power of some of the statistical comparisons, particularly between and among the female and male groupings and between the less prevalent GI and endocrine conditions; however, promising associations are present, as evident by the data in **Tables 3** and **4**. It is also possible that certain GI and endocrine conditions are underreported or are not documented in the medical record and thus not detected during study enrollment. Furthermore, the effect of medication on these markers is not known, although there were no significant differences in the use of specific therapeutics among the four GI and endocrine categories in this study. Also, previous studies in antipsychotic-naïve cohorts indicated that differences in marker levels between groups were not a function of medication (30, 31). However, other unidentified environmental or other confounders may contribute to these findings.

Our results indicate that the relationships between GI, endocrine and immune systems in schizophrenia are complex. It is possible that co-existing GI and endocrine pathologies in people with psychiatric disorders further compound inflammation and dysregulation of immune pathways, some of which may impact the brain. Understanding the biochemical mechanisms of how these imbalances in peripheral systems relate to cognitive deficits and psychiatric symptoms may lead to new strategies to more effectively prevent and treat these disorders.

## Data Availability Statement

The datasets generated for this study are available on request to the corresponding author and in compliance withinstitutional data sharing regulations.

## Ethics Statement

The studies involving human participants were reviewed and approved by the Institutional Review Boards (IRB) of the Sheppard Pratt Health System and the Johns Hopkins Medical Institution following established guidelines. This research was performed in accordance with The Code of Ethics of the World Medical Association (Declaration of Helsinki) for experiments involving humans. The patients/participants provided their written informed consent to participate in this study.

## Author Contributions

ES conceived the idea for the paper and performed the data analyses. ES, FD, and RY wrote the paper.

## Funding

This work was supported by a NIMH P50 Silvio O. Conte Center at Johns Hopkins (grant# MH-94268) and by the Stanley Medical Research Institute. The funding sources had no involvement in study design; collection, analysis and interpretation of data; in the writing of the report; and in the decision to submit the article for publication.

## Conflict of Interest

The authors declare that the research was conducted in the absence of any commercial or financial relationships that could be construed as a potential conflict of interest.

## References

[1] Pouget JG The Emerging Immunogenetic Architecture of Schizophrenia. Schizophr Bull (2018) 44:993–1004. 10.1093/schbul/sby038 29701842 PMC6135230

[2] Sekar A Bialas AR de Rivera H Davis A Hammond TR Kamitaki N Schizophrenia risk from complex variation of complement component 4. Nature (2016) 530:177–83. 10.1038/nature16549 PMC4752392 26814963

[3] Woo JJ Pouget JG Zai CC Kennedy JL The complement system in schizophrenia: where are we now and what’s next? Mol Psychiatry (2019) 25:114–30. 10.1038/s41380-019-0479-0 31439935

[4] Severance EG Yolken RH From Infection to the Microbiome: An Evolving Role of Microbes in Schizophrenia. Curr Top Behav Neurosci (2019) 44:67–84. 10.1007/7854_2018_84 PMC6732248 30847804

[5] Kohler O Petersen L Mors O Mortensen PB Yolken RH Gasse C Infections and exposure to anti-infective agents and the risk of severe mental disorders: a nationwide study. Acta Psychiatr Scand (2017) 135:97–105. 10.1111/acps.12671 27870529

[6] Bechter K Updating the mild encephalitis hypothesis of schizophrenia. Prog Neuropsychopharmacol Biol Psychiatry (2013) 42:71–91. 10.1016/j.pnpbp.2012.06.019 22765923

[7] Muller N Inflammation in Schizophrenia: Pathogenetic Aspects and Therapeutic Considerations. Schizophr Bull (2018) 44:973–82. 10.1093/schbul/sby024 PMC6101562 29648618

[8] Thaiss CA Zmora N Levy M Elinav E The microbiome and innate immunity. Nature (2016) 535:65–74. 10.1038/nature18847 27383981

[9] Honda K Littman DR The microbiota in adaptive immune homeostasis and disease. Nature (2016) 535:75–84. 10.1038/nature18848 27383982

[10] Severance EG Prandovszky E Castiglione J Yolken RH Gastroenterology issues in schizophrenia: why the gut matters. Curr Psychiatry Rep (2015) 17:27. 10.1007/s11920-015-0574-0 25773227 PMC4437570

[11] Liemburg EJ Nolte IM P. investigators Klein HC Knegtering H Relation of inflammatory markers with symptoms of psychotic disorders: a large cohort study. Prog Neuropsychopharmacol Biol Psychiatry (2018) 86:89–94. 10.1016/j.pnpbp.2018.04.006 29778547

[12] Radhakrishnan R Kaser M Guloksuz S The Link Between the Immune System, Environment, and Psychosis. Schizophr Bull (2017) 43:693–7. 10.1093/schbul/sbx057 PMC5472105 28969353

[13] Dumontaud M Korchia T Khouani J Lancon C Auquier P Boyer L Sexual dysfunctions in schizophrenia: Beyond antipsychotics. A systematic review. Prog Neuropsychopharmacol Biol Psychiatry (2019) 98:109804. 10.1016/j.pnpbp.2019.109804 31711954

[14] Sarsenbayeva A Marques-Santos CM Thombare K Di Nunzio G Almby KE Lundqvist M Effects of second-generation antipsychotics on human subcutaneous adipose tissue metabolism. Psychoneuroendocrinology (2019) 110:104445. 10.1016/j.psyneuen.2019.104445 31563732

[15] Zorn JV Schur RR Boks MP Kahn RS Joels M Vinkers CH Cortisol stress reactivity across psychiatric disorders: A systematic review and meta-analysis. Psychoneuroendocrinology (2017) 77:25–36. 10.1016/j.psyneuen.2016.11.036 28012291

[16] Romero-Sanchez M Gonzalez-Serna A Pacheco YM Ferrando-Martinez S Machmach K Garcia-Garcia M Different biological significance of sCD14 and LPS in HIV-infection: importance of the immunovirology stage and association with HIV-disease progression markers. J Infect (2012) 65:431–8. 10.1016/j.jinf.2012.06.008 22728172

[17] Weber NS Gressitt KL Cowan DN Niebuhr DW Yolken RH Severance EG Monocyte activation detected prior to a diagnosis of schizophrenia in the US Military New Onset Psychosis Project (MNOPP). Schizophr Res (2018) 197:465–9. 10.1016/j.schres.2017.12.016 PMC6033683 29310912

[18] Desplat-Jego S Johanet C Escande A Goetz J Fabien N Olsson N Update on Anti-Saccharomyces cerevisiae antibodies, anti-nuclear associated anti-neutrophil antibodies and antibodies to exocrine pancreas detected by indirect immunofluorescence as biomarkers in chronic inflammatory bowel diseases: results of a multicenter study. World J Gastroenterol : WJG (2007) 13:2312–8. 10.3748/wjg.v13.i16.2312 PMC4147139 17511029

[19] Kotze LM Nisihara RM Utiyama SR Kotze PG Theiss PM Olandoski M Antibodies anti-*Saccharomyces cerevisiae* (ASCA) do not differentiate Crohn’s disease from celiac disease. Arq Gastroenterol (2010) 47:242–5. 10.1590/S0004-28032010000300006 21140083

[20] Oshitani N Hato F Matsumoto T Jinno Y Sawa Y Hara J Decreased anti-*Saccharomyces cerevisiae* antibody titer by mesalazine in patients with Crohn’s disease. J Gastroenterol Hepatol (2000) 15:1400–3. 10.1046/j.1440-1746.2000.02357.x 11197050

[21] Catassi C Bai JC Bonaz B Bouma G Calabro A Carroccio A Non-Celiac Gluten sensitivity: the new frontier of gluten related disorders. Nutrients (2013) 5:3839–53. 10.3390/nu5103839 PMC3820047 24077239

[22] Leonard MM Sapone A Catassi C Fasano A Celiac Disease and Nonceliac Gluten Sensitivity: A Review. Jama (2017) 318:647–56. 10.1001/jama.2017.9730 28810029

[23] Dickerson F Origoni A Schroeder J Adamos M Katsafanas E Khushalani S Natural cause mortality in persons with serious mental illness. Acta Psychiatr Scand (2018) 137:371–9. 10.1111/acps.12880 29603145

[24] Dickerson F Stallings C Origoni A Katsafanas E Schweinfurth LA Savage CL Pentraxin 3 is reduced in bipolar disorder. Bipolar Disord (2015) 17:409–14. 10.1111/bdi.12281 25425421

[25] Dickerson F Stallings C Origoni A Vaughan C Khushalani S Yang S C-reactive protein is elevated in schizophrenia. Schizophr Res (2013) 143:198–202. 10.1016/j.schres.2012.10.041 23218564

[26] APA Diagnostic and statistical manual of mental disorders : DSM-IV-TR. Washington, DC: American Psychiatric Association (2000).

[27] Randolph C RBANS Manual - Repeatable Battery for the Assessment of Neuropsychological Status. San Antonio: Psychological Corporation (1998).

[28] Kay SR Fiszbein A Opler LA The positive and negative syndrome scale (PANSS) for schizophrenia. Schizophr Bull (1987) 13:261–76. 10.1093/schbul/13.2.261 3616518

[29] First MB Spitzer RL Gibbon M Williams JBW Structured Clinical Interview for DSM-IV Axis I Disorders - Non-patient Edition (SCID I/NP). New York: Biometrics Research, New York State Psychiatric Institute (1998).

[30] Severance EG Gressitt KL Stallings CR Origoni AE Khushalani S Leweke FM Discordant patterns of bacterial translocation markers and implications for innate immune imbalances in schizophrenia. Schizophr Res (2013) 148:130–7. 10.1016/j.schres.2013.05.018 PMC3732507 23746484

[31] Severance EG Alaedini A Yang S Halling M Gressitt KL Stallings CR Gastrointestinal inflammation and associated immune activation in schizophrenia. Schizophr Res (2012) 138:48–53. 10.1016/j.schres.2012.02.025 22446142 PMC4244845

[32] Severance EG Dickerson FB Halling M Krivogorsky B Haile L Yang S Subunit and whole molecule specificity of the anti-bovine casein immune response in recent onset psychosis and schizophrenia. Schizophr Res (2010) 118:240–7. 10.1016/j.schres.2009.12.030 20071146

[33] Severance EG Gressitt KL Halling M Stallings CR Origoni AE Vaughan C Complement C1q formation of immune complexes with milk caseins and wheat glutens in schizophrenia. Neurobiol Dis (2012) 48:447–53. 10.1016/j.nbd.2012.07.005 PMC3465075 22801085

[34] Dickerson F Stallings C Origoni A Vaughan C Khushalani S Leister F Markers of gluten sensitivity and celiac disease in recent-onset psychosis and multi-episode schizophrenia. Biol Psychiatry (2010) 68:100–4. 10.1016/j.biopsych.2010.03.021 20471632

[35] Cussotto S Sandhu KV Dinan TG Cryan JF The Neuroendocrinology of the Microbiota-Gut-Brain Axis: A Behavioural Perspective. Front Neuroendocrinol (2018) 51:80–101. 10.1016/j.yfrne.2018.04.002 29753796

[36] Jaggar M Rea K Spicak S Dinan TG Cryan JF You’ve Got Male: Sex and the Microbiota-Gut Brain Axis Across the Lifespan. Front Neuroendocrinol (2019) 56:100815. 10.1016/j.yfrne.2019.100815 31805290

[37] Kannan G Gressitt KL Yang S Stallings CR Katsafanas E Schweinfurth LA Pathogen-mediated NMDA receptor autoimmunity and cellular barrier dysfunction in schizophrenia. Transl Psychiatry (2017) 7:e1186. 10.1038/tp.2017.162 28763062 PMC5611729

[38] Severance EG Yolken RH Eaton WW Autoimmune diseases, gastrointestinal disorders and the microbiome in schizophrenia: more than a gut feeling. Schizophr Res (2016) 176:23–35. 10.1016/j.schres.2014.06.027 25034760 PMC4294997

